# Electrochemical Investigation of PANI:PPy/AC and PANI:PEDOT/AC Composites as Electrode Materials in Supercapacitors

**DOI:** 10.3390/polym14101976

**Published:** 2022-05-12

**Authors:** Shahbaz Khan, Mohammad Alkhedher, Rizwan Raza, Muhammad Ashfaq Ahmad, Abdul Majid, ElSayed M. Tag El Din

**Affiliations:** 1 Department of Physics, University of Gujrat, Gujrat 50700, Pakistan; shahbaz_khan@live.com; 2 Mechanical and Industrial Engineering Department, Abu Dhabi University, Abu Dhabi 111188, United Arab Emirates; mohammad.alkhedher@adu.ac.ae; 3 Clean Energy Research Lab (CERL), Department of Physics, COMSATS University Islamabad, Lahore Campus, Lahore 54000, Pakistan; rizwanraza@cuilahore.edu.pk (R.R.); maahmad@ciitlahore.edu.pk (M.A.A.); 4 Electrical Engineering Department, Faculty of Engineering & Technology, Future University in Egypt, New Cairo 11835, Egypt; elsayed.tageldin@fue.edu.eg

**Keywords:** activated carbon, conducting polymers, electrochemical properties, composite electrode, supercapacitors

## Abstract

The electrochemical deposition of the composites polyaniline (PANI):polypyrrole (PPy)/activated carbon (AC) and polyaniline (PANI): 3, 4-polyethylenedioxythiophene (PEDOT)/AC films is carried out in this work. The electrochemical character of the fabricated samples is investigated via cyclic voltammetry (CV), galvanostatic charge–discharge (GCD) and electrochemical impedance spectroscopy (EIS) using a three-electrode setup. The values of the specific capacitance of the composites PANI:PPy/AC and PANI:PEDOT/AC at a current density of 1 Ag^−1^ are evaluated as 586 Fg^−1^ and 611 Fg^−1^, respectively. The values of energy density are 40 Whkg^−1^ and 2094 Wkg^−1^, whereas power density is recorded as 44 Whkg^−1^ and 2160 Wkg^−1^ for respective composites PANI:PPy/AC and PANI:PEDOT/AC. Moreover, the respective composites appeared to retain cyclic stabilities of 92% and 90%. This study points to the potential of the prepared composites for application as electrodes in supercapacitors.

## 1. Introduction

The energy requirements for vehicles and portable electronic devices demand the preparation of improved energy storage systems that can sustain high energy density and power density [1,2,3,4,5,6]. Particularly, supercapacitors have attained special attention among other energy devices due to their capability of higher specific power and long cyclic life. The energy density (E) and power density (P) of supercapacitors may be enhanced by either increasing the capacitance or broadening the voltage window. The former can be established by employing electrodes with high capacitance, which has triggered extensive research activities to prepare new electrode materials. The popular electrode materials for supercapacitors include carbon species, conducting polymers and transition metal oxides [7,8,9]. Among transition metals, hydrous RuO_2_ has been considered a potential candidate as electrodes in supercapacitors, but its utilization on a commercial scale is not viable because of the high cost. Porous carbon and conducting polymers are cost-effective and environmentally friendly, which makes them suitable alternates of RuO_2_ [10,11,12,13]. Pore size and porosity are key features influencing the capacitance for supercapacitors in the case of porous carbon, which limit capacitance [14]. Further, the fabrication of biological cell templates of nitrogen-doped porous carbon and MnO_2_ composites that integrate biomass and energy, favoring an environmentally friendly strategy, has been reported [15]. Activated carbon (AC) is low cost and offers a high surface area among carbonaceous materials, due to which it is considered a potential candidate for supercapacitor electrodes. However, micro-pores in AC could not be effectively approached by electrolyte ions, and AC exhibits low electrical conductivity, which restricts its application in supercapacitors capable of optimum high power density [16].

The electrode materials based on conducting polymers are capable of fast, reversible redox reactions, which ensure high redox capacitance and high-density charge storage in energy devices [17,18,19,20,21]. The extensively studied conducting polymers for electrodes in supercapacitors are PPy, PANI and PEDOT [22,23]. Amongst the doped conducting polymers, doped PANI is more conductive and could be easily synthesized in aqueous solutions by electrochemical or chemical methods. The structure and morphology of PANI affect ions’ diffusivity and specific surface area, which in turn influences the redox behavior [24,25,26]. The experimental value of specific capacitance of PANI in H_2_SO_4_-based electrolytes has been observed in the range of 200–550 Fg^−1^ under a potential window of ~0.8 V. PANI is an active material transit between different oxidation states, and it stores charges by a redox reaction. However, the pseudocapacitive behavior of PANI involves shrinkage, swelling and cracking during the progression of doping/de-doping of charged ions that leads to poor cyclic stability. Moreover, the working potentials of PANI electrodes are high, which leads to their degradation at a relatively high potential and the occurrence of over-oxidation. This instability of PANI usually restricts its application as an energy material; however, its composite with active materials like carbon species and transition metal compounds may lessen such intrinsic drawbacks [27]. These issues have motivated the researchers to further explore composite materials based on electrochemical-friendly materials like carbon species and oxides of transition metals via the utilization of PANI as a matrix. 

PPy is preferred over other conducting polymers due to its cost-effectiveness, easy synthesis process in both aqueous and non-aqueous solutions, good stability and better electrical conductivity [28,29]. PPy in a doped state has exhibited specific capacitance in the range of 150–500 Fg^−1,^ whereas these values are sensitive to synthesis conditions and the morphology of the material [16,30,31,32,33,34,35]. The supercapacitive performance of the PPy electrode has been recorded using the CV technique in a 3 M KOH electrolyte, and a specific capacitance value of 931 F/g was evaluated at a 5 mV/s scan rate [36]. 

PEDOT exhibited good thermal stability, high chemical stability and high electrical conductivity values [37]. It has numerous benefits compared to other polythiophene derivatives because of the low oxidation potential, moderate energy band and better stability [38,39,40,41,42]. However, due to repeated oxidation and reduction, redox sites at the backbone of this polymer become no more stable, and ultimately, its cycling life becomes less when compared with carbonaceous electrodes [43]. As such, the capacitance and conductivity of carbon-based electrodes could be increased by combining them with CPs. Furthermore, composites of carbon and conducting polymers have been considered to enhance specific capacitance [44,45,46,47,48,49,50]. Moreover, it has been observed that conducting polymers’ thin films achieve better redox characteristics [51]. In order to benefit from the synergistic effect of good stability and capacitance, conducting polymers and carbon composites can be utilized in supercapacitors. This work is carried out with the aim of preparing new electrode materials based on composites comprising the electrochemically active constituents AC and one of copolymer PANI:PPy or PANI:PEDOT. The relevant properties of fabricated composites were comprehensively investigated using a variety of techniques.

## 2. Experimental

Materials, equipment and synthesis techniques are described in the following section.

### 2.1. Materials

AC, ANI 98%, Py 98%, EDOT 97%, LiClO_4_ 95.0% and 99% potassium ferricyanide (K_3_[Fe(CN)_6_]) from the Sigma Aldrich (Burlington, MA, USA) were purchased. Py and ANI were distilled prior to use in the experiment. The solutions were prepared in de-ionized water, and ITO glass (Sigma Aldrich, Burlington, MA, USA) was used as the substrate (working electrode).

### 2.2. Material Fabrication

**Figure 1:**
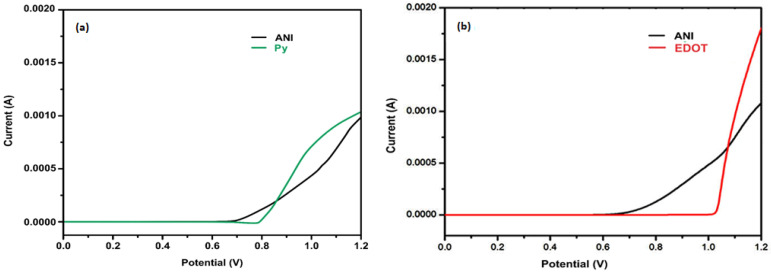
LSV curves (**a**) 10 mM Py and 10 mM ANI (**b**) 10 mM ANI and 10 mM EDOT in presence of 0.1 M LiClO_4_ on ITO, at potential range 0 to 1.2 V.

The electrochemical polymerization of composites was carried out using AUTOLABPGSTAT 12 (AUT73001, Artisan Technology Group, Kansas, MO, USA) inside a cell of three electrodes at room temperature, operating with GPES software. The ITO glass (1 cm^2^) was used as a working electrode, whereas the counter electrode was of platinum wire while Ag/AgCl served as a reference electrode to measure all potentials. The electrodes were washed ultrasonically for 15 minutes initially in acetone and then in ethanol, followed by cleaning with de-ionized water prior to the experiments. The composites PANI:PPy/AC and PANI:PEDOT/AC were synthesized electrochemically and deposited on the ITO by applying LSV intercept potential 0.85 V for PANI:PPy/AC and 1.07 V for PANI:PEDOT/AC up to 600 s. The LSV intercept point (potential) lies between the onset and maximum potential of monomers. Figure 1 illustrates the intercept point (potential) values for PANI:PPy/AC and PANI:PEDOT/AC, which are 0.85 V and 1.07 V, respectively. 

The composites films were fabricated by utilizing aqueous solutions of 10 mM of both monomers in 1:1 of each monomer (Py and ANI) for PANI:PPy/AC and (ANI and EDOT) for PANI:PEDOT/AC plus 0.1 M LiClO_4_ where AC particles were also dispersed in solution at the rate of 50 g·L^−1^. In order to avoid sedimentation of the AC particles during the electrochemical process, the solutions were agitated by purging purified nitrogen at 120 bubbles min^−1^.

## 3. Characterizations

The samples were characterized using FTIR for functional group analysis, SEM for morphology, BET for surface area, CV for specific capacitance and GCD for charging/discharging behavior. The results extracted on the basis of these characterizations are discussed below.

## 4. Results and Discussion

The analysis and results based on experimental observations to reveal the surface morphology, structural properties and electrochemical behavior of the synthesis films are discussed below.

### 4.1. FTIR Analysis

**Figure 2:**
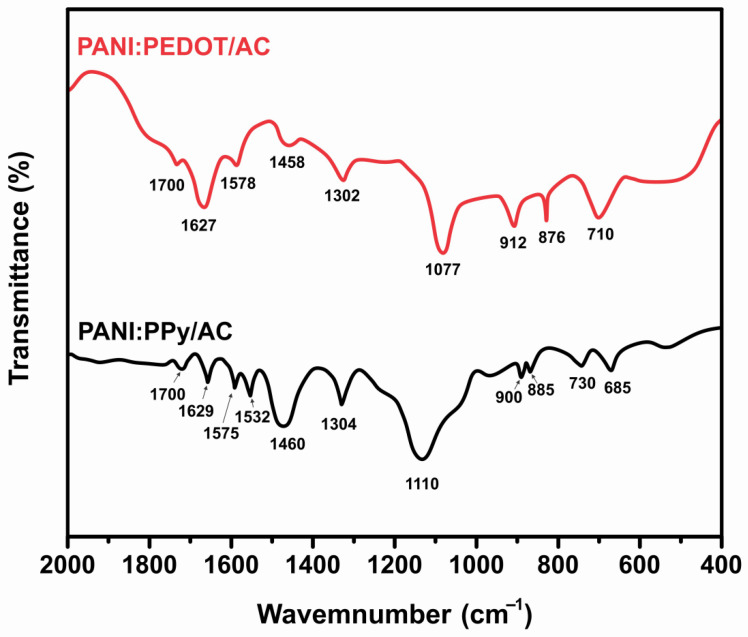
FTIR Spectra recorded for PANI:PPy/AC film and PANI:PEDOT/AC film.

Infrared spectra for composites PANI:PPy/AC and PANI:PEDOT/AC films were recorded to identify functional groups. The FTIR spectra of the composite films are presented in Figure 2. The spectra PANI:PPy/AC describe the first band at 685 cm^−1^, which is slightly shifted to a high wave number due to aniline phenosafranine copolymers and polyphenosafranine that caused stretching vibrations in C–H attributed to planer bending [52]. The sharp peaks located at 730 cm^−1^ and 900 cm^−1^ are assigned to the vibrations in =C–H, and these are associated with out-of-plane vibrations. Whereas in between these peaks, the addition of another sharp peak is found at 885 cm^−1,^ which confirms AC contents and presents functional group C–O–H. Further, a broader band appeared at 1110 cm^−1^ associated with N–H and owes to in-plane bending deformation [53]. A weaker hump before this band was assigned the C–H stretching due to in-plane bending of aromatic. In-plane stretching vibration is shown at 1304 cm^−1,^ whereas the existence of a benzene structure at 1460 cm^−1^ is also detected [42]. The band 700 cm^−1^ points to the presence of C=O due to the carboxylic group. The peaks at 1532 cm^−1^, 1575 cm^−1^ and 1629 cm^−1^ are due to C=C stretching associated with quinoid rings and aromatic group C=C, C=C stretching associated with benzenoid rings, respectively [54,55,56], whereas, the band at 1700 cm^−1^ revealed the presence of C=O due to a carboxylic group. 

The spectra for composite PANI:PEDOT/AC describes a bonding nature in which the peak 710 cm^−1^ is slightly shifted to a larger wave number owing to out-of-plane deformation in C–H [57]. The sharp peak located at 912 cm^−1^ is due to out-of-plane deformation in =C–H, whereas a peak detected at 876 cm^−1^ exhibits functional group C–O–H, which confirms the presence of AC content [58]. A weaker hump before this band assigned the C–H, which is in-plane bending of the aromatic group. The vibrations shown at 1302 cm^−1^ and 1458 cm^−1^ exhibit the presence of a benzene structure, whereas the band at 1700 cm^−1^ revealed the presence of C=O due to a carboxylic group. The bands at 1578 cm^−1^ and 1627 cm^−1^ are due to the C=C stretching vibration of the quinoid ring, aromatic group C=C, and C=C stretching vibration, pointing to the existence of a benzenoid ring [54]. 

### 4.2. Microstructure Analysis

**Figure 3:**
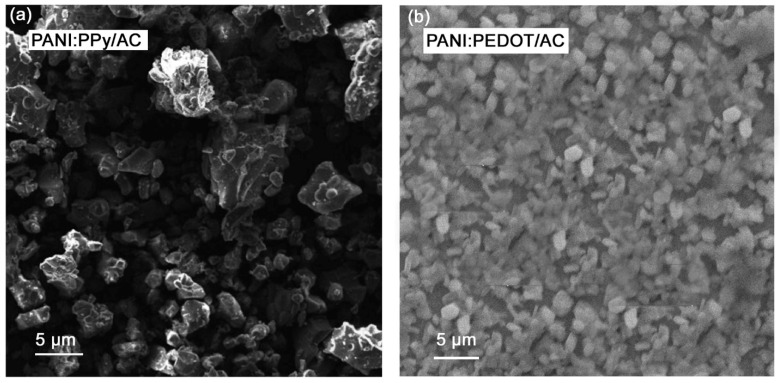
SEM images obtained for (**a**) PANI:PPy/AC and (**b**) PANI:PEDOT/AC films.

The properties of materials are highly influenced by the shape, size and morphology, due to which the prepared samples were investigated using SEM with the images shown in Figure 3a,b. It was observed that AC incorporation in PANI:PPy revealed interesting results in such a way that the surface area is increased due to smaller particle size, whereas porosity increases when individual polymers and copolymers are taken into account [59,60]. Moreover, AC incorporation in PANI:PPy appeared to cause fast charge carrier transportation, resulting in better kinetics.

The SEM image of PANI:PEDOT/AC is shown in Figure 3b, revealing the porous surface and exhibiting dark and bright phases. The dark phase plays an important role in the transportation of electrons, while the bright layer points to enhanced ionic transport, which are indicators of improvement in the overall performance of the supercapacitors [61,62]. The composite PANI:PEDOT/AC appeared to have uniform particle distribution with homogenous particles [55,57]. 

### 4.3. Surface Area Measurement and Pore Size Distribution

**Figure 4:**
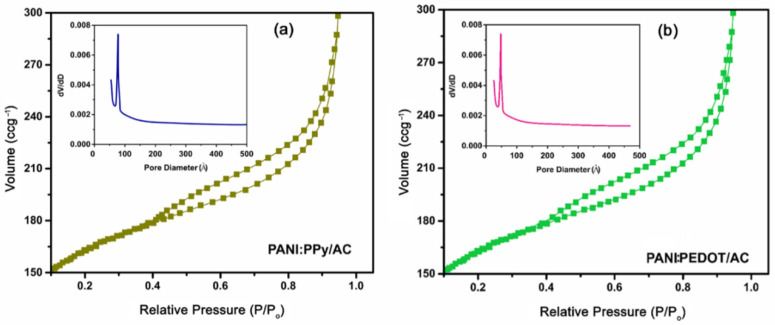
Isotherm of nitrogen adsorption–desorption recorded for (**a**) PANI:PPy/AC and (**b**) PANI:PEDOT/AC composite.

Figure 4a,b show nitrogen adsorption–desorption isotherm for PANI:PPy/AC and composite PANI:PEDOT/AC thin films deposited on ITO. The results revealed the mesoporous characteristics of the synthesized composites. 

A high value of the specific surface areas of 991 m^2^g^−1^ and 1021 m^2^g^−1^ was evaluated for the composite PANI:PPy/AC and PANI:PEDOT/AC, respectively. The high surface area for fabricated composites is due to the fact that PANI:PPy and PANI:PEDOT copolymers possess low-density values and attachment of the components PANI, PPy, PEDOT and AC in the composites. The porosity of PANI:PPy/AC and PANI:PEDOT/AC composites were also calculated using nitrogen adsorption–desorption isotherms. Moreover, pore sizes were evaluated as 7.3 nm (73 Å) and 4.89 nm (48.9 Å) for PANI:PPy/AC and PANI:PEDOT/AC, respectively, which are in the mesopore range (i.e., 2~50 nm).

### 4.4. Cyclic Voltammetry

**Figure 5:**
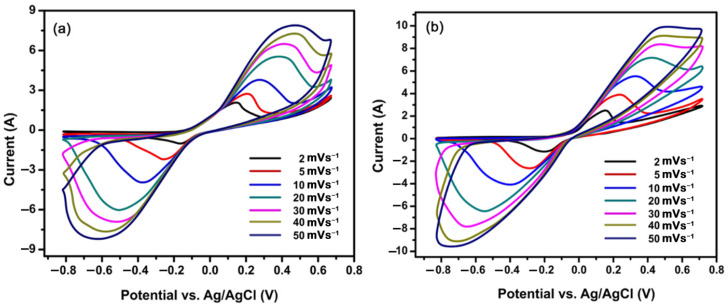
CVs measured of (**a**) PANI:PPy/AC and (**b**) PANI:PEDOT/AC in 2 M KCl.

Cyclic Voltammetry (CV) analyses of PANI:PPy/AC and PANI:PEDOT/AC composites were performed in 2 M KCl for different scan rates, including 2, 5, 10, 20, 30, 40 and 50 mVs^−1^. The CV of PANI:PPy/AC and PANI:PEDOT/AC composites exhibited typical pseudocapacitive performance, as shown in Figure 5a,b. The PANI:PPy/AC composite exhibited CV with a distinct oxidation/reduction reaction in a larger potential window with a value of 1.5 V (−0.8 to 0.7 V), as given in Figure 5a. It exhibited a value of capacitance as 583 Fg^−1^ scanned at a rate of 2 mVs^−1^. The composite PANI:PEDOT/AC exhibited CV with a distinct oxidation/reduction peak in a potential window with a value of 1.26 V (−0.73–0.53 V), as given in Figure 5b. It revealed a higher value of capacitance as 634 Fg^−1^ scanned at 2 mVs^−1^ when compared with composite PANI:PPy/AC.

**Figure 6:**
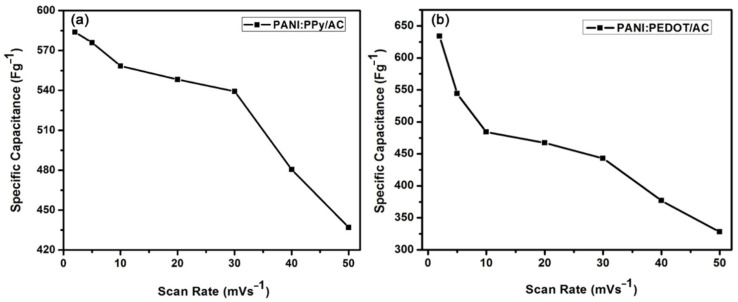
Specific capacitance versus scan rate recorded for (**a**) PANI:PPy/AC and (**b**) PANI:PEDOT/AC.

The well-defined oxidation peak and reduction peak occurrence at different scan rates exposed that the charging mechanism is Faradic and expresses pseudocapacitive performance in PANI:PPy/AC and PANI:PEDOT/AC composites. It is well established that an increase in scan rates results in enhancement in the area under the curve. Furthermore, the peaks of potential appeared to shift with the increase in values of the scan rate. The oxidation/reduction process occurred at the electrolyte/electrode interface, and the results confirm the pseudocapacitive behavior [63,64,65]. The specific capacitances (*C_s_*) of PANI:PPy/AC and PANI:PEDOT/AC composite films were calculated from CVs by applying Equation (1) [66].

(1) $$C_{s}=\frac{1}{m*S*\Delta V}\overset{V_{a}}{\underset{V_{c}}{\int }}IdV$$

where *m* represents active mass, and *S* is for scan rate. Here, Δ*V* represents a potential window, *I* is current, and *dV* represents potential during the Faradic reaction. The values of specific capacitances of composites PANI:PPy/AC and PANI:PEDOT/AC were 583 Fg^−1^ and 634 Fg^−1^, respectively, higher than the reported composites of conducting polymers and activated carbon, synthesized by electrochemical polymerization techniques [67,68,69]. Moreover, specific capacitance plots versus scan rate graphs for PANI:PPy/AC and PANI:PEDOT/AC composites in KCl electrolytes are given in Figure 6a,b.

### 4.5. Galvanostatic Charge/Discharge

**Figure 7:**
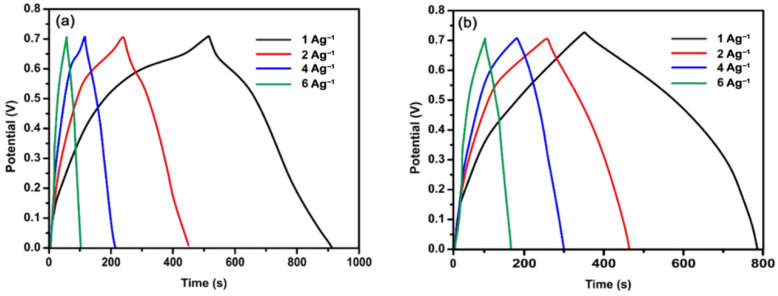
Galvanostatic charge/discharge measured for (**a**) PANI:PPy/AC and (**b**) PANI:PEDOT/AC in 2 M KCl.

Synthesized composites were characterized for analysis of charging and discharging behavior in a 2 M aqueous solution of KCl. The discharge times of composites PANI:PPy/AC and PANI:PEDOT/AC by applying a current density of 1 Ag^−1^ were observed as 410 s and 440 s, respectively, as per curves shown in Figure 7a,b. Moreover, it was observed that the value of discharge varies inversely with the value of applied current density, as elaborated in Figure 7a,b. It may be because, at a smaller current density, ions of electrolytes acquire sufficient time to access active material. Hence, the ions may face less resistance for lower excitation of the ions and vice versa. 

The calculation of specific capacitances (*C_s_*) of the composites PANI:PPy/AC and PANI:PEDOT/AC was carried out using GCD by applying Equation (2) [66,67].

(2) $$C_{s}=\frac{Idt}{mdV}$$

where *t* represents discharge time, *I* is applied current, *V* expresses the value of potential and m stands for active mass. The specific capacitances for PANI:PPy/AC are evaluated to be 586, 571, 514 and 428 Fg^−1^ by applying respective values of the current density 1, 2, 4 and 6 Ag^−1^. The calculated values of capacitance for PANI:PEDOT/AC are 611, 583, 556 and 500 Fg^−1^ at the respective values of the current density. The larger, exposed discharge plateau at a smaller, applied current density value has a fine correlation to the higher value of specific capacitance. In addition to this, our evaluated specific capacitance was considerably larger when compared with the reported values for conducting polymers/AC composites. The higher values of capacitance are assigned to a high value of electrical conductivity along with a larger volume of pores and a higher value of the surface area for the composites [67,68,69]. Thus, the measured capacitances for the prepared composites are basically ascribed to an increased area of the composite/solution interface like that of carbon materials and conducting polymers [68,69,70]. The composite films exhibit two charge/discharge trends, i.e., EDLC due to AC particles/solution boundary and a pseudo capacitor trend because of the conducting polymer [71,72,73]. 

The introduction of AC particles in copolymers PANI:PPy and PANI:PEDOT to produce the composites PANI:PPy/AC and PANI:PEDOT/AC appeared to support the requisite redox character. The electrochemical properties of the composites are mainly linked with AC and the presence of an oxygen functional group at the surface. Moreover, electrical conductivity for AC does not depend on potential, so its incorporation in the copolymer films is expected to contribute to conductivity in the composites. It has been investigated that the incorporation of AC particles in the polymer films increases porosity as well as surface area, which appeared to support the penetration of Cl^−^ into the composite films during the doping process [74]. 

**Figure 8:**
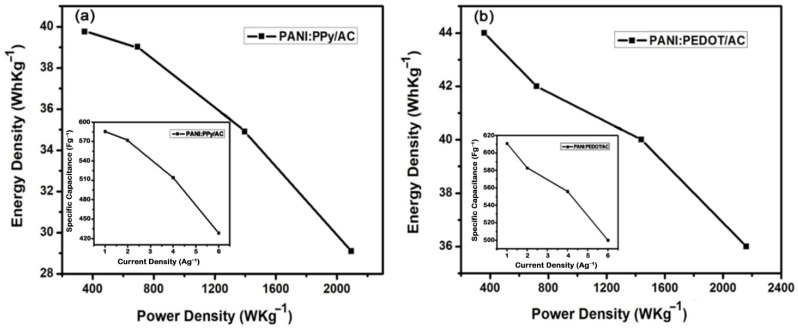
Ragone plot exhibiting energy density versus power density for (**a**) PANI:PPy/AC and (**b**) PANI:PEDOT/AC.

The supercapacitors’ most substantial parameters, like E and P, were calculated using galvanostatic discharge behavior using Equations (3) and (4) [66,75]. Moreover, the Ragone plot for energy density versus power density has been drawn and shown in Figure 8.

(3) $$E=\frac{1}{2}C_{s\;}{\left(\Delta V\right)}^{2}$$

(4) $$P=\frac{E}{t}$$

where *C_s_* denotes specific capacitance, Δ*V* refers to the potential window and *P* gives power density. Further, energy density and discharge time are represented by *E* and *t,* respectively.

The Ragone plot for PANI:PPy/AC is illustrated in Figure 8a, according to which the value of energy density varied from 40–29 WhKg^−1^, and the value of power density is found in the range of 349–2094 WKg^−1^ at the current density values of 1, 2, 4 and 6 Ag^−1^ as listed in Table 1. The high value of the energy density points to a larger value of electrical conductivity, which is a consequence of high pore volume and the enhanced value of the surface area for PANI:PPy/AC.

The Ragone plot drawn for the composite PANI:PEDOT/AC is shown in Figure 8b, which indicates values of energy density in the range of 44–36 WhKg^−1^, and power density in the range of 360–2161 WKg^−1^ at the current density values of 1, 2, 4 and 6 Ag^−1^ as given in Table 2. The reasonably higher values of energy density point to high electrical conductivity, which owe to high pore volume and enhanced value of the surface area for PANI:PEDOT/AC composite [76,77].

### 4.6. Electrochemical Impedance Spectroscopy

In order to investigate the electrochemical character of composites PANI:PPy/AC and PANI:PEDOT/AC, the samples were analyzed using potentiostat/galvanostat under the application of a perturbation ac signal of 5 mV within the frequency range 0.1 Hz–10^5^ Hz. The Nyquist plot is drawn to exhibit real and imaginary parts to indicate the frequency response of the composites within a 5 mM solution of K_3_ [Fe(CN)_6_], including a 0.1 M solution of KCl as a supporting electrolyte.

**Figure 9:**
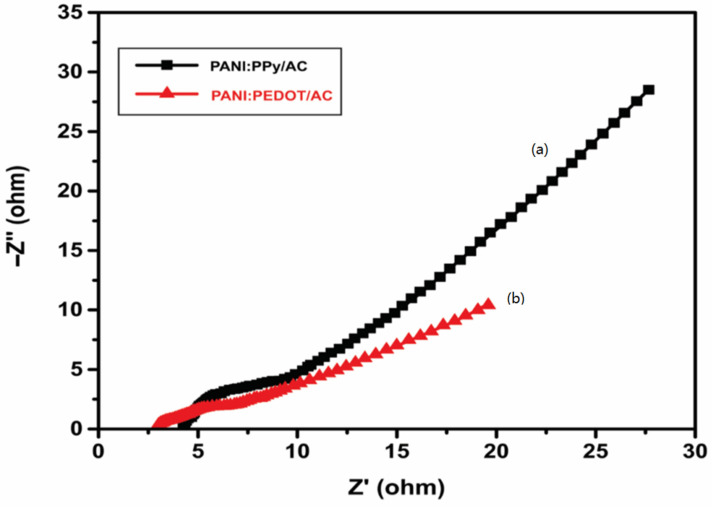
Nyquist plots of (**a**) PANI:PPy /AC composite and (**b**) PANI:PEDOT/AC composite within 5 mM of K_3_ [Fe(CN)_6_] plus 0.1 M KCl.

The Nyquist plots for PANI:PPy/AC and PANI:PEDOT/AC composites are elaborated in Figure 9a,b, respectively. The plots show a quasi-semicircle-like trend in the high-frequency region and a linear trend in the low-frequency region. The intercept of the arc of the quasi-semicircle on the real axis in a high-frequency region corresponds to the value of R_s_ (electrolyte/electrode interface resistance) [78]. The straight line starting point on the real axis matches R_ct_ (within electrodes resistance faced by charge transfer). The evaluated values of R_s_ and R_ct_ for composite PANI;PPy/AC are 4.5 Ω and 8 Ω, respectively. However, R_s_ and R_ct_ for composite PANI:PEDOT/AC are 3.1 Ω and 5 Ω, respectively. Moreover, smaller R_s_ and R_ct_ values indicate the rapid transfer of ions in the electrolyte and diffusion on the surface of the electrode. The Nyquist plot of two prepared electrodes revealed significant improvement in values of R_s_ and R_ct_ [79,80]. Moreover, the straight lines for two composites in the low-frequency region sloped at 45°, indicating the smooth diffusion of ions [81].

### 4.7. Cyclic Stability

PANI and PPy composite-based electrodes are known to retain specific capacitance below 50% of the initial value up to continuous cycling for 1000 cycles. Hence, in the case of the conducting polymer composites, cycling instability is considered a major barrier in practical applications of the electrodes in supercapacitors. For the purpose of improving the cycling stability of conducting polymer composites, several strategies have been developed. Wang et al. observed that PANI deposition on the surface of coral-like monolithic carbon film can attain a higher value of capacitance up to 78.2% after 1000 cycles [82]. Hai et al. observed that carbon nanotube incorporation in PPy nanowires retains 85% specific capacitance from its initial value after 1000 cycles [83]. Zhang et al. demonstrated that deposition of PANI and PPy on a substrate of reduced graphene oxide accomplished respective values of capacitance retention up to 82% and 81% when tested for 1000 cycles [84]. Shahbaz et al. reported that PEDOT:PPy/AC deposition on ITO substrate retains 87.5% specific capacitance up to 10,000 cycles [85]. The comparison of cyclic stability for different reported composites within this study is given in Table 3.

**Figure 10:**
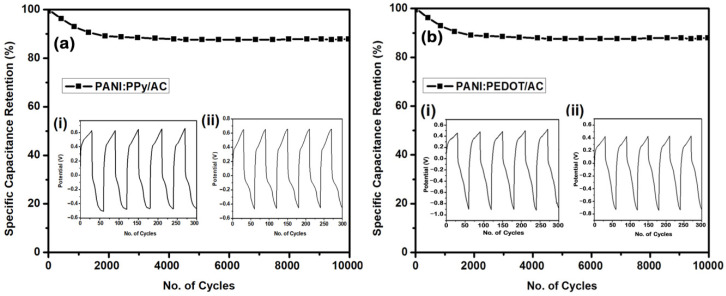
Cyclic stability curve of PANI:PPy/AC and PANI:PEDOT/AC composite for 10,000 cycles. The inset shows the first ((**a**,**b**) (**i**)) and last five cycles ((**a**,**b**) (**ii**)) for the composites.

In this study, the prepared composites electrodes PANI:PPy/AC and PANI:PEDOT/AC were tested for cycling stability at a current density of 1 Ag^−1^ after continuous cycling for 10,000 cycles, as presented in Figure 10. It was found that the value of specific capacitance retention for PANI:PPy/AC and PANI:PEDOT/AC appeared to be 92% and 90%, respectively, when compared with the initial values. The measured, high values of specific capacitance retentions are assigned to the synergistic effect of PANI, PPy, PEDOT and AC in the composites [83,84,85,93]. Recently, self-assembled nanostructures of NiS/carbon have been tested for hybrid supercapacitors [94].

## 5. Summary

The films of the composites PANI:PPy/AC and PANI:PEDOT/AC were deposited on ITO glass using electrochemical polymerization. The CVs demonstrated that the composite electrodes exhibit pseudocapacitive characteristics. The specific capacitance values for PANI:PPy/AC and PANI:PEDOT/AC evaluated by galvanostatic discharge appeared as 586 Fg^−1^ and 611 Fg^−1^ at 1 Ag^−1^. The GCD exhibited discharge times of 410 s and 440 s for PANI:PPy/AC and PANI:PEDOT/AC, respectively, whereas the composites exhibited a considerably high value of energy density of 40 WhKg^−1^ and 44 WhKg^−1,^ respectively. The value of power densities for PANI:PPy/AC composite and PANI:PEDOT/AC composite are evaluated as 2094 WKg^−1^ and 2160 WKg^−1,^ respectively, at a current density of 6 Ag^−1^. The electrode stability for the prepared composites was also evaluated, which exhibited specific capacitance retention at 92% for PANI:PPy/AC and 90% for PANI:PEDOT/AC after cycling up to 10,000 cycles. The observed high specific capacitance along with energy density demonstrates the potential of PANI:PPy/AC and PANI:PEDOT/AC films as suitable electrode materials for supercapacitor applications. The findings of this study revealed the synergistic benefits and excellent electrochemical behavior of the studied composites for energy storage applications.

## Figures and Tables

**Table 1:** Energy density and power density measured for the samples at different values of current density for PANI:PPy/AC composite film deposited on ITO Glass.

Current Density (Ag^−1^)	Specific Capacitance (Fg^−1^)	Energy Density (Whkg^−1^)	Power Density (Wkg^−1^)
1	586	40	349
2	571	39	698
4	514	35	1396
6	429	29	2094

**Table 2:** Energy density and power density measured for the samples at different values of current densities for PANI:PEDOT/AC composite film deposition on ITO Glass.

Current Density (Ag^−1^)	Specific Capacitance (Fg^−1^)	Energy Density (Whkg^−1^)	Power Density (Wkg^−1^)
1	611	44	360
2	583	42	720
4	556	40	1440
6	500	36	2160

**Table 3:** Synthesis methods and electrochemical performance of reported PANI-based composites as supercapacitors electrode materials.

Composite	Synthesis Technique	Specific Capacitance	Cyclic Stability	References
PANI/Porous Carbon	electrochemical polymerization	180 Fg^−1^	91% after 1000 cycles	[86]
PANI/MWCNTS	chemical oxidative polymerization	320 Fg^−1^	8% after 50 cycles	[49]
GRAPHENE/PANI NANOFIBER	in situ polymerization	480 Fg^−1^	70% after 1000 cycles	[87]
GO/PANI	a soft chemical route	531 Fg^−1^	N/A	[88]
PANI/CUO	in situ polymerization	286.35 Fg^−1^	N/A	[89]
RUO_2_/PANI	in situ oxidative polymerization	425 Fg^−1^	N/A	[90]
PANI/Fe_3_O_4_	in situ polymerization	572 Fg^−1^	82% over 5000 cycles	[91]
SNO_2_/PANI	in situ oxidative polymerization	335.5 Fg^−1^	99% after 1000 cycles	[92]
PANI/AC	electrochemical polymerization	200 Fg^−1^	N/A	[67]
PANI:PEDOT	electrochemical polymerization	0.62 mFcm^−2^	N/A	[57]
PANI:PPy	electrochemical polymerization	227 Fg^−1^	N/A	[69]
PANI:PPy/AC	electrochemical polymerization	586 Fg^−1^	92% after 10,000 cycles	*
PANI:PEDOT/AC	electrochemical polymerization	611 Fg^−1^	90% after 10,000 cycles	*

* This work.

## Data Availability

Not applicable.
